# Academia steps up to strengthen health policymaking in Romania: a roadmap for tackling difficulties encountered by Ministry of Health specialty commissions

**DOI:** 10.25122/jml-2021-1005

**Published:** 2021

**Authors:** Dafin Muresanu, Bogdan Popescu, Stefan Strilciuc, Constantin Radu, Cristina Tiu

**Affiliations:** 1. Romanian Neurology Specialty Commission, Ministry of Health, Bucharest, Romania; 2. Department of Neuroscience, Iuliu Hatieganu University of Medicine and Pharmacy, Cluj-Napoca, Romania; 3. RoNeuro Institute for Neurological Research and Diagnostic, Cluj-Napoca, Romania; 4. Department of Neurology, Colentina Clinical Hospital, Bucharest, Romania; 5. Department of Clinical Neurosciences, Carol Davila University of Medicine and Pharmacy, Bucharest, Romania; 6. Stroke Unit, Department of Neurology, University Emergency Hospital, Bucharest, Romania

## Background

The Romanian Ministry of Health (MoH) appoints specialty commissions (SCs), usually constituted by the most prominent clinicians nationwide, to handle various essential tasks, ranging from issuing clinical guidelines to developing training curricula for resident doctors or answering specific requests from the minister of health and other health officials [1]. Together, they provide the MoH with the necessary scientific expertise to operate the health system, serving as core pillars of the policymaking process and significantly contributing to shaping the healthcare landscape at the national level [2].

While there is not much literature to describe the intricate processes of devising health policies in Romania, nor any robust indicators for evaluating MoH activity, a general impression of low administrative and technical capacity is consistently rendered throughout the grapevine. An epiphenomenon of said undercapacity is the overall scanty performance of our health system, as shown by several reports, perhaps most notably by Romania’s OECD Country Health Profile [3]. Notwithstanding our country’s problem with human resources in healthcare, health information systems have been historically unable to provide essential information for SCs, particularly epidemiological data, performance indicators for national health programs, and expenditure information [4]. Being tasked to generate evidence-based strategies that substantiate national health policy, SC activity is also impeded by the lack of a practical information highway between health institutions. Moreover, the MoH has not yet developed sufficient capacity to provide proper logistical support to facilitate the work of the SCs (e.g., digital support is limited to a video communications platform used for remote sessions). However, even while facing such shortcomings, members of all SCs strive to fulfill their duties at their utmost best. With management functions falling almost entirely on the commissions themselves, academic representatives have pooled resources and organizational skills to create work environments that fit their specific needs. We dare assert that the plethora of assignments dealt with by each commission benefit from almost no coordination, expertise, or support from their leading institutional counterparts.

In this editorial, we report what we believe has been a quintessentially constructive experience in an overall dismal environment of Romanian public health governance. While our experience is highlighted with this occasion, this report is by no means an exhaustive account of SC success stories or other accomplishments that have emerged from the academic crucible. Nor do we aim to rank and appraise the performance of SCs. Instead, our intention is primarily to showcase good practices we have come across and emphasize the need for additional support from our political leaders.

## The case of the Romanian Neurology Specialty Commission

Using resources outside the MoH or other key institutions such as the National Health Insurance House (NHIH) and relying on pro-bono contributions from members of academia, notably the Romanian Society of Neurology - the country’s most prestigious professional body in the field - the Romanian Neurology Specialty Commission (RNSC) has managed in recent years to promote developments on several levels. As in the case of many related entities in our country, this multidimensional growth was kindled and later spearheaded by the late professor Ovidiu Alexandru Bajenaru, an influencing neurologist whose powerful impact on our field will likely drive positive change for many years to come [5].

In terms of workload, the RNSC responded to 32 requests during 2020, related to a variety of areas and topics:

• Developing, substantiating, and providing updates for various health services, therapeutic protocols, and health programs operated by the MoH and NHIH;
• Defining and verifying compliance with the criteria for infrastructure and human resources involved in the national health programs;
• Evaluating various proposals to extend patients’ access to medical services (e.g., multiple sclerosis treatment centers);
• Various queries related to medicines and medical devices (e.g., listing strategic drugs, providing expert opinions of off-label use of drugs, establishing the value-added tax status of medical devices, substantiating special needs authorizations etc);
• Support for cost-volume contracts (e.g., defining eligible population groups, treatment comparators, drug priority criteria etc);
• Providing clinical expertise and responding to individual patient petitions;
• Addressing the neurological complications of COVID-19 and contributing to the immunization campaign.

Due to the pandemic, SC workloads have increased significantly. To promote a reduction in response times to certain important issues on the ministerial agenda without additional costs from the state budget, the RNSC has managed to assemble a team of outstanding clinicians that ensure the representativeness of each region of Romania and all branches of neurology. Neurology is a complex medical specialty. The diversity of requests to the RNSC requires multidisciplinary expertise in a wide range of clinical subspecialties.

Therefore, the expansion approach also aimed to consolidate the expertise of this group. The SCs are often asked for opinions on the local infrastructure, as well as on the epidemiological distribution in the centers that provide medical services on the Romanian territory. Through this initiative, we also aimed to increase the representativeness and geographical coverage of the commission. For all these reasons, since November 2020, the RNSC has started organizing human resources by the following areas of competence and corresponding expert groups:

• Cerebrovascular Diseases;
• Autonomic Nervous System Disorders;
• Epilepsy;
• Multiple Sclerosis and Demyelinating Disorders;
• Neurodegenerative Diseases;
• Neurorehabilitation;
• Neurosonology;
• Neuro-Oncology;
• Parkinson’s disease;
• Peripheral Nervous System Disorders;
• Rare Neurological Disorders;
• Sleep Disorders.

To facilitate the process of elaboration, adjustment, and validation of the answers, the group collaborates via a project management platform (Basecamp), which allows easy communication between members, access to and storage of working documents, as well as active consultations, ensuring thus the efficiency and transparency of the consultative process. Nevertheless, the paradigm shift extends beyond internal organizational aspects. Given the importance of neurological and neurology-related pathology, our community will have an important role to play in consulting and implementing the new vision of the European Union. In our view, alignment with European standards involves, among other things:

• Elaboration of coherent strategies for the development of infrastructure and human resources, which integrate the needs of all diseases and the prevention area, in a transdisciplinary manner;
• Drafting clinical guidelines with rigorous methodology (e.g., *Grading of Recommendations Assessment, Development and Evaluation – GRADE*);
• Building neurological expertise for the Health Technology Assessment, a notoriously underdeveloped area in our country;
• Efficient transfer of innovation from other countries to Romania.

The RNSC is currently working to develop a five-year master plan for target group populations to be used as a benchmark for cost-volume contracts (CVCs). This project is essential for ensuring continued and improved access to medicines for diseases such as multiple sclerosis, which rely on such contracts in the national health programs. In the absence of comprehensive disease registries and with very little robust epidemiological data on hand, the RNSC plans to establish reference sources for compiling target group estimates. This will allow negotiating entities means to obtain financial agreements that are more affordable for the Romanian healthcare system while providing transparency and predictability for drug producers and retailers. While there are many other problems associated with CVCs (e.g., they are unsuitable for innovative interventions such as cell and gene therapy), SCs have no power to influence risk-sharing agreements at the national level. Therefore, our initiative is targeted at optimizing the financing of CVC molecules within the current legal constraints.

All previously stated objectives are completely aligned with the mission of the new working group formed at the level of the Romanian Society of Neurology. It is imperative that through our collective strength and competence, we unite all efforts to promote the interests of clinicians, patients, and researchers, thus aligning with the needs of the authorities, and especially the challenges faced by the health system.

## Conclusions and recommendations

Due to the breadth of legal responsibilities, SCs are often faced with rather bureaucratic requests that do not best use their members’ shared knowledge and expertise. Such tasks could instead be undertaken by administrative entities of the Ministry or other health authorities.

Unquestionably, the Romanian MoH must carry out extensive tasks under tremendous pressure, especially considering the COVID-19 pandemic. Presently, every bit of effort must be invested into keeping the healthcare system able to treat both COVID and non-COVID patients, and the specialty commissions are working tirelessly to help accomplish this endeavor. While the current situation leaves little room for comprehensive institutional reforms, we must begin laying the groundwork for a future in which the potential of the specialty commissions is used to its fullest extent.

The advancement opportunities brought by our National Recovery and Resilience Plan must not only be reflected in physical health infrastructure but should also determine rethinking core aspects of health policymaking. A more precise set of attributions, more organizational and logistical support are some of the steps the MoH could take to harvest more of the consultative capacity of the specialty commissions. Just as the RNSC would promptly respond to an invitation to participate in reshaping the role and increasing the effectiveness of these consultative bodies, certainly other SCs would too. Given the health needs of our country, as pointed out by the above-cited reports, conceiving national plans that tackle the leading public health issues in Romania is an absolute priority, to which SCs and the Romanian Society of Neurology could contribute extensively.

Daring further, SCs could one day become an autonomous body for scientific consultation and appraisal in health, such as the National Institute for Health and Care Excellence (NICE) in the United Kingdom. A professional body of this kind could fill essential gaps in the Romanian healthcare system, covering essential but underserved areas such as Health Technology Assessment. This way, the SCs may reach their full potential and fulfill their role of bridging evidence and decision-making in health.

## References

[1] Capitolul II – Comisiile de specialitate - Ordinul nr. 1202/2017 privind înfiinţarea, organizarea şi funcţionarea comisiilor şi subcomisiilor de specialitate ale Ministerului Sănătăţii. https://lege5.ro/Gratuit/ge4dqmjug4zq/comisiile-de-specialitate-ordin-1202-2017?dp=giytgmbrgmyteoa.

[2] Vladescu C, Scintee SG, Olsavszky V, Hernandez-Quevedo C, Sagan A (2016). Romania: Health System Review. Health Syst Transit.

[3] OECD/European Observatory on Health Systems and Policies (2019), Romania: Country Health Profile 2019, State of Health in the EU

[4] Strilciuc S, Done N, Gheorghe A (2017). Budgets revealed: drilling down through Romania’s hospital expenditure: Stefan Strilciuc. European Journal of Public Health.

[5] Muresanu D (2020). In memoriam: Professor Ovidiu Alexandru Bajenaru (1957-2020). J Med Life 2020.

